# Quantitative Proteomic Analysis Reveals Unfolded-Protein Response Involved in Severe Fever with Thrombocytopenia Syndrome Virus Infection

**DOI:** 10.1128/JVI.00308-19

**Published:** 2019-05-01

**Authors:** Lei-Ke Zhang, Bo Wang, Qilin Xin, Weijuan Shang, Shu Shen, Gengfu Xiao, Fei Deng, Hualin Wang, Zhihong Hu, Manli Wang

**Affiliations:** a State Key Laboratory of Virology, Wuhan Institute of Virology, Chinese Academy of Sciences, Wuhan, People’s Republic of China; b University of Chinese Academy of Sciences, Beijing, People’s Republic of China; University of Kentucky College of Medicine

**Keywords:** ATF6, ER stress, PERK, SFTSV, XBP1, quantitative proteomics, unfolded-protein response, virus-host interaction

## Abstract

Severe fever with thrombocytopenia syndrome (SFTS) is an emerging, highly pathogenic, infectious disease caused by infection with a newly discovered tick-borne phlebovirus, SFTS virus (SFTSV). Limited information on the molecular mechanism of SFTSV infection and pathogenesis impedes the development of effective vaccines and drugs for SFTS prevention and treatment. In this study, an isobaric tag for relative and absolute quantification (iTRAQ)-based quantitative proteomic analysis of SFTSV-infected HEK 293 cells was performed to explore dynamic host cellular protein responses toward SFTSV infection. A total of 433 of 5,606 host proteins involved in different biological processes were differentially regulated by SFTSV infection. The proteomic results highlighted a potential role of endoplasmic reticular stress-triggered unfolded-protein response (UPR) in SFTSV infection. Further functional studies confirmed that all three major branches of the UPR, including the PKR-like endoplasmic reticulum kinase (PERK), the activating transcription factor-6 (ATF6), and the inositol-requiring protein-1 (IRE1)/X-box-binding protein 1 (XBP1) pathways, were activated by SFTSV. However, only the former two pathways play a crucial role in SFTSV infection. Furthermore, expression of SFTSV glycoprotein (GP) alone was sufficient to stimulate the UPR, whereas suppression of PERK and ATF6 notably decreased GP expression. Interestingly, two other newly discovered phleboviruses, Heartland virus and Guertu virus, also stimulated the UPR, suggesting a common mechanism shared by these genetically related phleboviruses. This study provides a global view to our knowledge on how host cells respond to SFTSV infection and highlights that host cell UPR plays an important role in phlebovirus infection.

**IMPORTANCE** Severe fever with thrombocytopenia syndrome virus (SFTSV) is an emerging tick-borne bunyavirus that causes severe fever with thrombocytopenia syndrome in humans, with a mortality rate reaching up to 30% in some outbreaks. There are currently no U.S. Food and Drug Administration-approved vaccines or specific antivirals available against SFTSV. To comprehensively understand the molecular interactions occurring between SFTSV and the host cell, we exploit quantitative proteomic approach to investigate the dynamic host cellular responses to SFTSV infection. The results highlight multiple biological processes being regulated by SFTSV infection. Among these, we focused on exploration of the mechanism of how SFTSV infection stimulates the host cell’s unfolded-protein response (UPR) and identified the UPR as a common feature shared by SFTSV-related new emerging phleboviruses. This study, for the first time to our knowledge, provides a global map for host cellular responses to SFTSV infection and highlighted potential host targets for further research.

## Abstract (precis)

Severe fever with thrombocytopenia syndrome virus (SFTSV) is an emerging tick-borne bunyavirus that causes severe fever with thrombocytopenia syndrome in humans, with a mortality rate reaching up to 30% in some outbreaks. There are currently no U.S. Food and Drug Administration-approved vaccines or specific antivirals available against SFTSV. To comprehensively understand the molecular interactions occurring between SFTSV and the host cell, we exploit quantitative proteomic approach to investigate the dynamic host cellular responses to SFTSV infection. The results highlight multiple biological processes being regulated by SFTSV infection. Among these, we focused on exploration of the mechanism of how SFTSV infection stimulates the host cell’s unfolded-protein response (UPR) and identified the UPR as a common feature shared by SFTSV-related new emerging phleboviruses. This study, for the first time to our knowledge, provides a global map for host cellular responses to SFTSV infection and highlighted potential host targets for further research.

## INTRODUCTION

Severe fever with thrombocytopenia syndrome virus (SFTSV) is an emerging tick-borne virus that causes severe fever with thrombocytopenia syndrome (SFTS). Since the first report in China in 2009, SFTSV has spread over China, South Korea, and Japan, with a mortality rate reaching up to 30% (1–4). SFTSV is a novel member of the genus *Phlebovirus*, family *Phenuiviridae*, order *Bunyavirales* (https://talk.ictvonline.org/taxonomy). Recently, a succession of other novel emerging phleboviruses that are closely related to SFTSV, including Heartland virus (HRTV; identified in the United States in 2012) (5), Hunter island group virus (HRGV; identified in Australia in 2014) (6), and Guertu virus (GTV; identified in China in 2018) (7), have been reported, highlighting their potential threats to public health. Currently, there are no therapeutics or US Food and Drug Administration (FDA)-approved vaccines to combat infections of SFTSV and these related viruses.

SFTSV is an enveloped virus with a tripartite, single-stranded, negative-sense RNA genome comprising large (L), middle (M), and small (S) segments. The L segment encodes an RNA-dependent RNA polymerase (RdRP), while the M segment encodes glycoproteins Gn and Gc, which form a heterodimer on the surface of the virus particle to mediate viral entry and egress. The S segment employs an ambisense strategy to encode nucleoprotein (NP) and nonstructural protein (NSs). SFTSV infection is initiated by virus binding to cell attachment factors, including C-type lectins and nonmuscle myosin heavy chain IIA, followed by internalization of virions into clathrin-mediated endocytosis (8). After the release of viral ribonucleoprotein in the cytoplasm, replication and transcription of viral genomes start. The assembly and release of SFTSV progeny virions occur at the Golgi apparatus and Golgi-derived vesicles. To establish successful infection, SFTSV must manipulate host proteins to favor its own replication. However, there lacks a comprehensive understanding of the molecular interactions occurring between SFTSV and host cells (9).

Virus infection induces different stress responses in host cells. The endoplasmic reticulum (ER) stress response is a highly conserved mechanism that may arise from accumulation of misfolded or unfolded proteins, depletion of ER membranes for virus assembly and release, competition with host proteins for modifications by viral glycoproteins, etc. (10). To relieve ER stress and reestablish protein folding homeostasis, a series of intracellular protein quality control signaling pathways known as the unfolded-protein response (UPR) are activated. The UPR induces cellular transcriptional and translational responses, resulting in global inhibition of protein synthesis to reduce protein overload, upregulation of molecular chaperones to promote protein folding, as well as activation of ER-associated degradation (ERAD) to eliminate unfolded proteins from the ER (11). The UPR is regulated by three main signaling branches, namely, the PKR-like endoplasmic reticulum kinase (PERK) (12), the activating transcription factor-6 (ATF6) (13), and the inositol-requiring protein-1 (IRE1)/X-box-binding protein 1 (XBP1) (14) pathways. Many viruses, including both enveloped viruses (herpesviruses, flaviviruses, coronaviruses, arenaviruses, etc.) and nonenveloped viruses (coxsackievirus), can trigger ER stress and the UPR during their infections. In many cases, activation of the UPR is required for efficient virus replication (15, 16). For example, the arenavirus lymphocytic choriomeningitis virus, which is also a negative-sense, single-stranded RNA virus with a segmented genome, activates the ATF6 pathway for optimal virus multiplication during acute infection (17). In contrast, a recent report showed that an alphacoronavirus transmissible gastroenteritis virus (TGEV) infection induced ER stress and triggered the UPR, and ER stress negatively regulated TGEV replication (18). Activation of the UPR also contributes to virus pathogenesis. Another recent study indicated that Zika virus (ZIKV) infection triggered the UPR in the cerebral cortex of infected postmortem human fetuses, which disturbed normal neurogenesis and contributed to ZIKV-associated microcephaly (19). It has been reported that Tula hantavirus triggers proapoptotic signals of ER stress in Vero E6 cells (20). However, the detailed interactions between bunyaviruses and three main signaling branches of the UPR have not been reported yet.

In this study, to systematically identify host proteins involved in SFTSV-host interactions, the dynamic host cellular responses to SFTSV infection were investigated by isobaric tag for relative and absolute quantification (iTRAQ)-based quantitative proteomic analysis. iTRAQ is an isobaric labeling method employed in quantitative proteomics by tandem mass spectrometry (MS/MS) for the identification and quantitation of proteins from different sources in a single experiment (20, 21). Human embryonic kidney 293 (HEK 293) cells are highly permissive to SFTSV infection, and an *in vivo* model showed that SFTSV replicated and caused pathological changes or lesions in kidney cells in mice and macaques (22, 23). In addition, a wide variety of functional studies of SFTSV have been performed in this cell line, so we decided to choose HEK 293 cells for proteomic study (7, 24, 25). Our results provide a global map showing how host cells respond to SFTSV infection and highlight multiple biological processes being regulated by SFTSV infection. Among these, we focused on exploration of the mechanism of how SFTSV infection stimulates host cell UPR and, in turn, how the three classical pathways of UPR affect SFTSV infection.

## RESULTS

### Global host cellular protein responses to SFTSV infection revealed by quantitative proteomic analysis.

**FIG 1:**
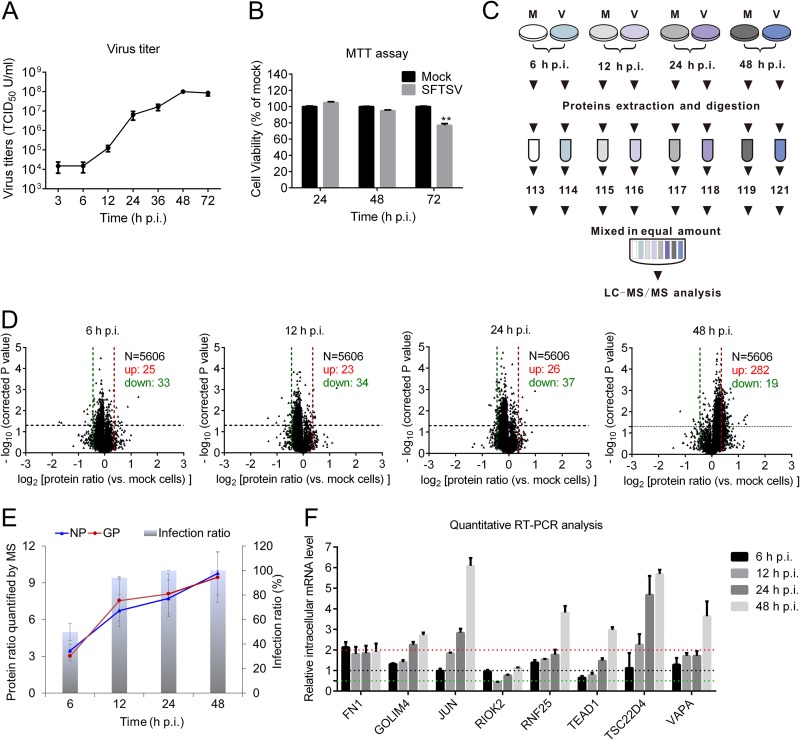
Quantitative proteomics analysis of SFTSV-infected HEK 293 cells. (A) Kinetics of SFTSV replication in HEK 293 cells. HEK 293 cells were infected with SFTSV at an MOI of 5, the supernatants were harvested at the indicated time points, and the virus titers were measured by determining the TCID_50_. All experiments were performed at least three times, and values represent means ± the SDs from three replicates. (B) HEK 293 cells were infected with SFTSV at an MOI of 5, and at the indicated time points HEK 293 cells were harvested and subjected to MTT assay to measure cell viability. (C) Workflow for iTRAQ-based quantitative proteomic analysis of SFTSV-infected HEK 293 cells. (D) Volcano plot showing log_2_-fold change plotted against the –log_2_-adjusted *P* value for SFTSV-infected cells versus mock-treated cells at different times p.i. (E) Kinetics of the viral protein ratio and infection ratio of SFTSV-infected HEK 293 cells. The viral protein ratio was measured by MS. The infection ratio was measured by detecting NP-positive cells versus all cells detected. (F) Validation of MS results using quantitative RT-PCR. HEK 293 cells were infected with SFTSV at an MOI of 5 or mock infected. At indicated time intervals, cells were harvested, and intracellular mRNAs were extracted and subjected to reverse transcription. The intracellular RNA levels of the corresponding proteins were measured with quantitative RT-PCR. Glyceraldehyde-3-phosphate dehydrogenase (GAPDH), tubulin, and actin were chosen as internal controls. Intracellular RNA levels at each time point of SFTSV infection were normalized to those in the mock-infected cells. The experiments were repeated twice. Bars in panels A and D represent the SD. M, mock treated; V, SFTSV infected; N, number of proteins quantified; up, upregulated protein; down, downregulated protein; FN1, fibronectin; GOLIM4, Golgi integral membrane protein 4; JUN, transcription factor AP-1; RIOK2, serine/threonine protein kinase RIO2; RNF25, E3 ubiquitin-protein ligase RNF25; TEAD1, transcriptional enhancer factor TEF-1; TSC22D4, TSC22 domain family protein 4; VAPA, vesicle-associated membrane protein-associated protein A.

Before quantitative proteomic analysis, the growth kinetics of SFTSV in HEK 293 cells was monitored by measuring viral titer. Briefly, HEK 293 cells were infected with SFTSV at a multiplicity of infectivity (MOI) of 5, and the viral titers were measured with endpoint dilution assays (EPDAs). As shown in Fig. 1A, the replication rate of SFTSV increased over the time from 6 to 48 h postinfection (p.i.), while after 48 h p.i., the replication of SFTSV entered into stationary phase. The highest virus titer could reach ∼1 × 10^8^ 50% tissue culture infectious doses (TCID_50_)/cell at 48 h p.i., which is consistent with a recent report (7). An MTT [3-(4,5-dimethyl-2-thiazolyl)-2,5-diphenyl-2*H*-tetrazolium bromide] assay showed that SFTSV had no significant effect on cell viability before 48 h p.i. (Fig. 1B). Therefore, samples collected at 6, 12, 24, and 48 h p.i. were used for iTRAQ-based quantitative proteomic analysis. As illustrated in Fig. 1C, the extracted proteins from SFTSV-infected or mock-infected cells were subjected to trypsin digestion and further labeled with different iTRAQ reagents. The peptides were then mixed at a ratio of 1 and subjected to strong cation exchange chromatography fractionation and liquid chromatography MS/MS (LC-MS/MS) analysis. Three independent biological replicates were performed at all four time points.

As a result, a total of 5,606 host proteins were quantified (see Table S1 in the supplemental material). The ratio distributions of all quantified proteins were profiled (Fig. 1D). Among the identified host proteins, 25 were upregulated and 33 were downregulated at 6 h p.i., 23 were upregulated and 34 were downregulated at 12 h p.i., 26 were upregulated and 37 were downregulated at 24 h p.i., and 282 were upregulated and 19 were downregulated at 48 h p.i. Differentially expressed proteins were significantly enriched at 48 h p.i., suggesting that SFTSV replication had a profound impact on host cells at this time point. Further examination of these proteins indicated that 26 host proteins were differentially regulated at two time points. Seven host proteins were differentially regulated at three time points, including cytochrome *c* oxidase assembly protein COX16 homolog (COX16), FUN14 domain-containing protein 1 (FUNDC1), E3 ubiquitin-protein ligase TRIM21 (TRIM21), vacuolar protein sorting-associated protein 13D (VPS13D), endoplasmic reticulum chaperone BiP (GRP78), basement membrane-specific heparan sulfate proteoglycan core protein (HSPG2), and zinc finger and BTB domain-containing protein 7A (ZBTB7A), while fibronectin (FN1) was upregulated at all four time points (Table S2). To find out the possible relationship between virus infection with host cellular responses, we further determined virus infection ratio and the expression patterns of the major viral proteins (the nucleocapsid protein [NP] and the glycoprotein [GP]). Approximately 49.98, 94.37, 99.96, and 100% cells were infected (Fig. 1E, gray columns), and approximately 3-, 7-, 8-, and 10-fold increases occurred in the NP and GP protein levels (Fig. 1E, blue and red lines) at 6, 12, 24, and 48 h p.i., respectively. Therefore, compared to the kinetics of virus infection and viral protein expression, the host protein changes to SFTSV infection seemed to be delayed and modest.

To validate our MS data, quantitative real-time-PCR (RT-PCR) analysis to determine the transcription levels of eight randomly selected host proteins was performed. Although the fold changes quantified by quantitative RT-PCR and MS were not identical, their change tendencies were similar. As shown in Fig. 1F, quantitative RT-PCR data indicated that genes encoding FN1, Golgi integral membrane protein 4 (GOLIM), transcription factor AP-1 (JUN), E3 ubiquitin-protein ligase RNF25 (RNF25), transcriptional enhancer factor TEF-1 (TEAD1), TSC22 domain family protein 4 (TSC22D4), and vesicle-associated membrane protein-associated protein A (VAPA) were upregulated, whereas the gene encoding serine/threonine-protein kinase RIO2 (RIOK2) was first downregulated and then recovered as a result of SFTSV infection, which was consistent with our MS data (Table S1).

**FIG 2:**
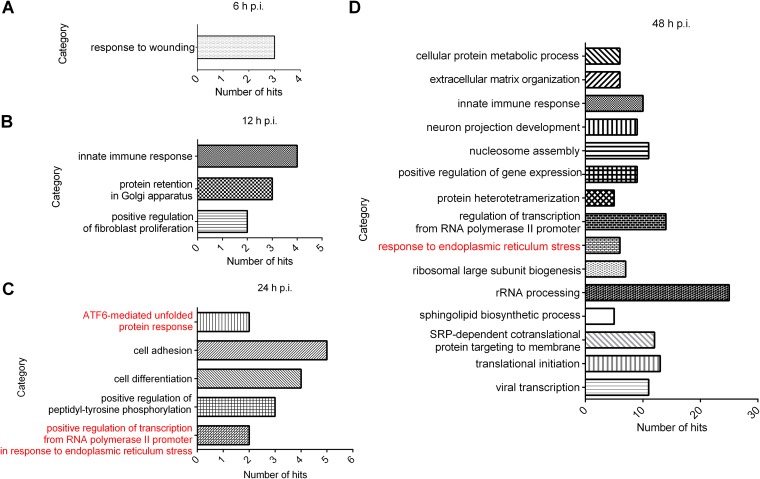
GO analysis of regulated proteins based on biological processes. Differentially regulated proteins at each time point were subjected to DAVID, respectively. Regulated proteins were grouped based on their roles in biological processes, and a statistical overrepresentation test was performed to determine which biological process was overrepresented by differentially regulated proteins. Only biological processes overrepresented by differentially regulated proteins at 6 h p.i. (A), 12 h p.i. (B), 24 h p.i. (C), and 48 h p.i. (D) were considered to be regulated by SFTSV infection. The categories labeled in red in panels C and D are UPR-related pathways.

To determine which biological processes were regulated during the SFTSV infection process, gene ontology (GO) analysis was performed. As shown in Fig. 2, at 6 h p.i., only the process “response to wounding” was overrepresented (Fig. 2A), while at 12 h p.i., “positive regulation of fibroblast proliferation,” “protein retention in Golgi apparatus,” and “innate immune response” were overrepresented (Fig. 2B). At 24 h p.i., five biological processes were found to be upregulated and two of them (“ATF6-mediated unfold protein response” and “positive regulation of transcription from RNA polymerase II promoter in response to endoplasmic reticulum stress”) belong to ER stress response (Fig. 2C). More biological processes were overrepresented at 48 h p.i., and again the “response to endoplasmic reticulum stress” was upregulated and six hits from the category were significantly enriched (Fig. 2D). These data revealed a prominent cluster of upregulated host genes linked to ER stress and highlighted that ER stress response was triggered by SFTSV infection. Since the role of the UPR in bunyavirus life cycle has not been thoroughly depicted, we therefore decided to perform an in-depth analysis on this biological process.

### UPR is activated by SFTSV infection.

**FIG 3:**
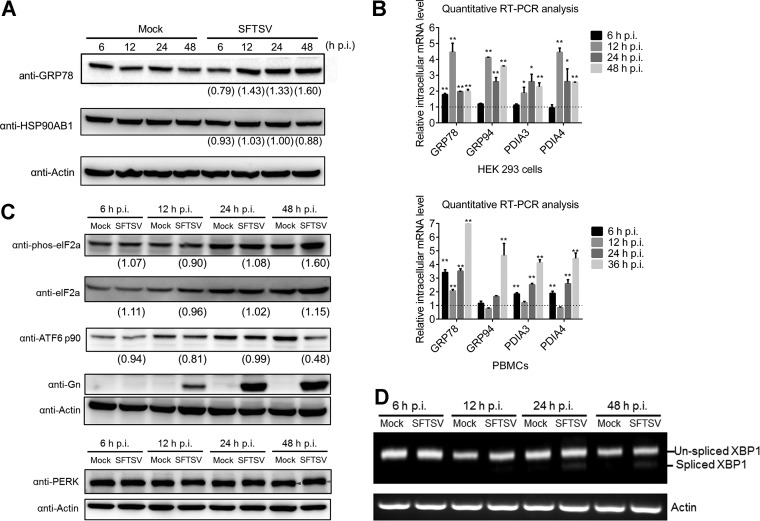
SFTSV infection activates all three branches of the UPR. (A) SFTSV-infected HEK 293 cells were collected at the indicated time intervals, and total proteins were extracted and subjected to Western blot analysis for GRP78/94. (B) RNA samples from the above cells were extracted and subjected to reverse transcription. The relative mRNA levels of the indicated proteins were measured using quantitative RT-PCR. GAPDH, tubulin, and actin were chosen as internal controls. Intracellular RNA levels at each time point of SFTSV infection were normalized to those in the mock-infected cells. All experiments were performed at least three times, and values represent means ± the SDs from three replicates. *, *P* < 0.05; **, *P* < 0.01 (Student *t* test). (C) SFTSV-infected HEK 293 cells were collected at the indicated time intervals, and total proteins were extracted and subjected to Western blot analysis for ATF6 p90, phos-eIF2α (Ser51), total eIF2α, PERK, Gn, and the internal control actin. Red arrow, phosphorylated PERK; purple arrow, PERK. (D) RNA samples were also analyzed for spliced *XBP1* mRNA by using reverse transcription-PCR. The intensity of protein band was measured by ImageJ_v1.8.0. For each time point, the protein intensity was first normalized to actin and then normalized to the corresponding mock group.

One consequence of activating ER stress is the upregulation of genes involved in protein folding, such as ER chaperones, as represented by GRP78 and glucose related protein 94 (GRP94), and isomerases, including protein disulfide isomerase A3 and A4 (PDIA3 and PDIA4). Therefore, Western blot and quantitative RT-PCR analyses were performed to determine whether these ER chaperones were regulated during SFTSV infection. As shown in Fig. 3A, intracellular protein levels of GRP78 were upregulated at 12, 24, and 48 h p.i., while as a control the intracellular protein levels of a cytosolic chaperone, HSP90AB1, was not upregulated. We further found that mRNA levels of *GRP78/GRP94*, as well as *PDIA3/PDIA4*, increased after SFTSV infection (Fig. 3B), confirming that ER stress was activated by SFTSV infection in HEK 293 cells. To better reflect the situation of virus infection *in vivo*, mouse peripheral blood mononuclear cells (PBMCs) were infected with SFTSV at an MOI of 5 and subjected to quantitative RT-PCR analysis. We found that the intracellular mRNA levels of *GRP78*, *GRP94*, *PDIA3*, and *PDIA4* were elevated significantly in SFTSV-infected PBMCs, indicating that the ER stress response was activated as a result of SFTSV infection in PBMCs (Fig. 3B).

UPR is a cellular adaptive response for restoring ER homeostasis in response to ER stress (11). Here, we detected the effects of SFTSV infection on three branches of the UPR, including the PERK, ATF6, and IRE1 pathways. PERK is an ER-localized kinase whose lumenal domain senses an excess of unfolded proteins that enter the ER (26). Here, Western blot analysis of HEK 293 cell extracts indicated PERK was phosphorylated at 48 h p.i. in SFTSV-infected cells (Fig. 3C), suggesting that PERK was activated. Activated PERK will further phosphorylate the α subunit of eukaryotic initiation factor 2 (eIF2α) and thus attenuate global protein synthesis. We therefore investigated the phosphorylation state of eIF2α during SFTSV infection over a 48-h time course and found a significant increase in the levels of phosphorylated eIF2α, but not of the total eIF2α proteins, at 48 h p.i. in SFTSV-infected cells compared to the mock-infected group (Fig. 3C), suggesting that the PERK pathway is activated upon SFTSV infection. Next, the activation of the ATF6 signaling pathway during SFTSV infection was examined. ATF6 is constitutively expressed as a 90-kDa protein (namely, ATF6 p90), and upon ER stress the ATF6 is cleaved to an N-terminal 50-kDa protein (namely, ATF6 p50). Western blot analysis showed that ATF6 was decreased at 48 h p.i. (Fig. 3C), suggesting that the ATF6 signaling pathway was activated at the late phase of SFTSV infection. Viral GP was used to indicate successful infection (Fig. 3C). Finally, the activation of the IRE1-XBP1 pathway was examined. Activation of IRE1 causes posttranscriptional cleavage of the *XBP1* mRNA (unspliced *XBP1*) that produces the spliced form of *XBP1* mRNA, which encodes the transcriptionally active form of the *XBP1* gene. Similarly, HEK 293 cells were mock treated or infected with SFTSV over a 48-h time course. Both unspliced *XBP1* and spliced *XBP1* were amplified by reverse transcription-PCR. As shown in Fig. 3D, spliced *XBP1* was detected at 24 and 48 h p.i. in SFTSV-infected cells but not in mock-infected cells, suggesting that the IRE1 pathway was also activated by SFTSV at these time points. These results demonstrated that SFTSV infection could activate all three classical branches of the UPR.

### PERK and ATF6 signaling pathways play critical roles in SFTSV infection processes.

**FIG 4:**
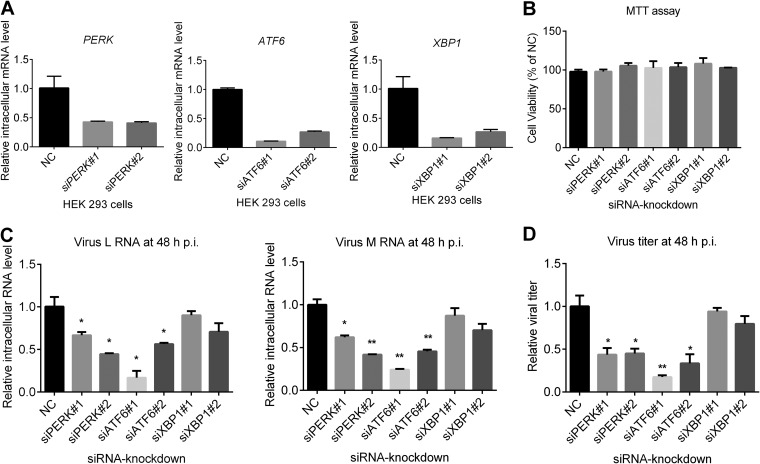
Effects of UPR on SFTSV production. (A and B) Knockdown of targeted proteins by using RNA interference. HEK 293 cells were transfected with siRNAs against targeted host genes or scrambled siRNA (NC). Cells were collected at 48 h p.t., and total cellular RNA was extracted and subjected to reverse transcription. Intracellular RNA levels of *ATF6*, *XBP1*, and *PERK* were measured with quantitative RT-PCR (A). MTT analysis was performed to determine the CPE of the siRNAs. (B). (C and D) Effects of knockdown of host proteins on SFTSV production and replication. HEK 293 cells were transfected with siRNAs against targeted genes or scrambled siRNA (NC), and at 48 h p.t. the cells were superinfected with SFTSV at an MOI of 1. (C) At 48 h p.i., total cellular RNA was extracted, and SFTSV genomic RNA levels were measured with quantitative RT-PCR. (D) The cell supernatant was collected, and the viral titer was measured by EPDAs. All experiments were performed in triplicate, and values represent means ± the SDs from three replicates. *, *P* < 0.05; **, *P* < 0.01 (Student *t* test).

The upregulation of the UPR in SFTSV-infected cells implied that the UPR might play a role in viral replication. Thus, we explored the roles of three branches of the UPR in the SFTSV replication process by RNA interference (RNAi). Briefly, HEK 293 cells were transfected with small interfering RNA (siRNA) targeting *PERK*, *ATF6*, and *XBP1*, or scrambled siRNA, and the knockdown efficiency was detected at 24 h posttransfection (p.t.) by quantitative RT-PCR (Fig. 4A). All siRNAs used could reduce intracellular mRNA levels of *XBP1*, *ATF6*, and *PERK* significantly without cytopathic effects (CPE) (Fig. 4A and B). At 24 h after siRNA transfection, the cells were infected with SFTSV at an MOI of 1 and were also collected at 48 h p.i. Quantitative RT-PCR results showed that the intracellular levels of viral RNA (L and M segment) were lower in ATF6 or PERK knockdown cells but not in XBP1 knockdown cells (Fig. 4C), indicating that depletion of ATF6 or PERK inhibited SFTSV replication. The supernatant was also collected at 48 h p.i., and viral titers were determined. As shown in Fig. 4D, SFTSV titers were significantly decreased in ATF6 or PERK knockdown cells but not in XBP-1 silenced cells, a finding similar to the result of Fig. 4C. These results indicated that two of the three main branches of the UPR, the ATF6 and PERK pathways, facilitated SFTSV infection process.

### Expression of SFTSV GP upregulates the UPR.

**FIG 5:**
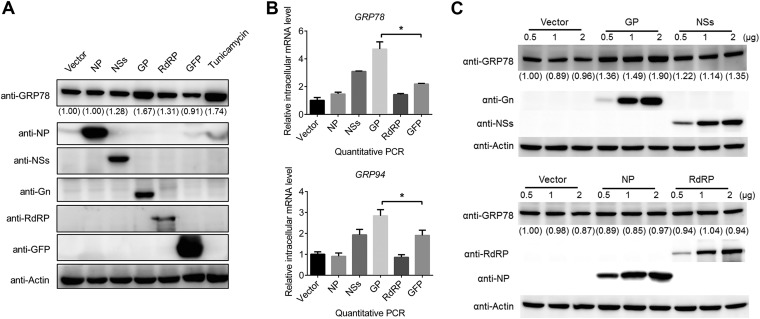
Expression of SFTSV G proteins, but not other viral proteins, induces the cellular UPR. (A) HEK 293 cells were transfected with plasmids expressing viral proteins, or with GFP or empty vector as controls, and at 48 h p.t. the cells were collected, and the intracellular protein levels of GRP94, GRP78, viral proteins, and the loading control actin were detected with Western blots. Tunicamycin (Tm), a reported inducer of the UPR, was used as a positive control. (B) HEK 293 cells were transfected with plasmids expressing viral proteins or GFP, and the intracellular RNA levels of *GRP94* and *GRP78* were detected with quantitative RT-PCR at 48 h p.t. (C) HEK 293 cells were transfected with increasing amounts of SFTSV protein-expressing plasmids and empty vector as a control. At 48 h p.t., the cells were collected, and the intracellular protein levels of GRP78, viral proteins, and actin as a loading control were detected by Western blotting. *, *P* < 0.05; **, *P* < 0.01 (Student *t* test). The intensity of protein band was measured by ImageJ_v1.8.0. Protein intensity was first normalized to actin and then further normalized to that of the empty vector-transfected cells.

To explore how SFTSV triggers the UPR, the effect of expression of individual SFTSV proteins on the UPR was examined. HEK 293 cells were transfected with empty vector, or vectors expressing different SFTSV proteins or GFP as a control. At 48 h p.t., the expression of viral proteins and GFP in HEK 293 cells was confirmed by Western blot analyses (Fig. 5A). Then, the intracellular protein levels of GRP78 and GRP94 were further determined by Western blot analysis. As shown in Fig. 5A, GRP78 and GRP94 increased only in cells expressing GP (the full-length glycoprotein, containing both Gn and Gc) or cells treated with tunicamycin (Tm), a reported inducer of the UPR, but not in cells expressing other viral proteins, including NP, NSs, and RdRP, or the control GFP. We also detected intracellular mRNA levels of *GRP78* and *GRP94* in HEK 293 cells by quantitative RT-PCR. As shown in Fig. 5B, the intracellular mRNA levels of *GRP78* and *GRP94* were significantly elevated in GP-expressing cells. We further found that compared to other viral proteins or empty vector control, GP could activate the expression of GRP78 in a dose-dependent manner (Fig. 5C). These results suggested that the SFTSV GP alone can activate the UPR.

### PERK and ATF6 signaling pathways are important for maintaining intracellular levels of GP.

**FIG 6:**
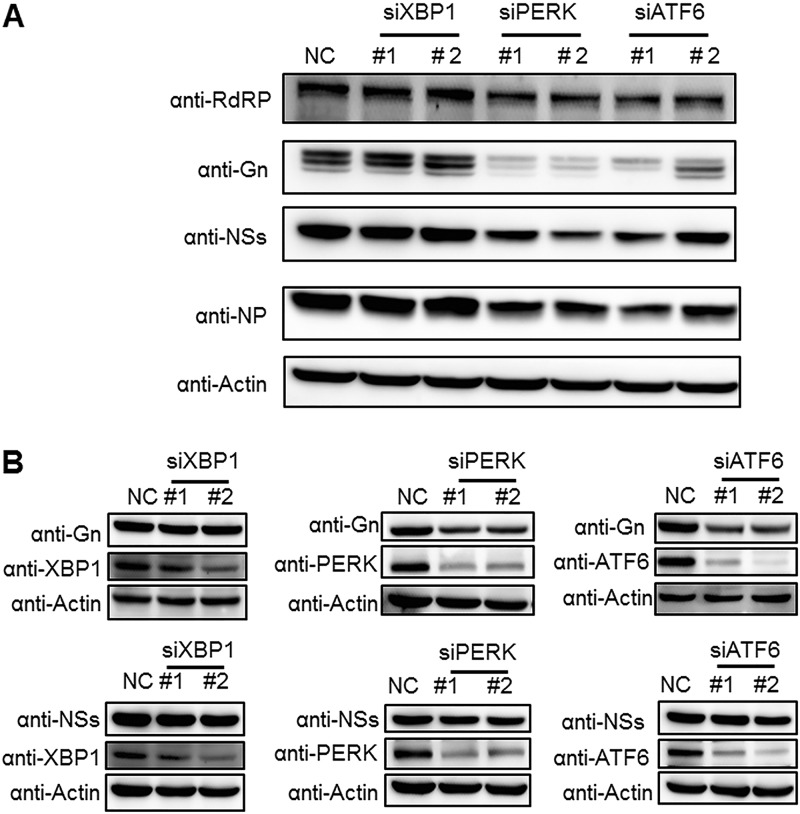
Knockdown of PERK and ATF6 reduces intracellular levels of SFTSV GP. (A) HEK 293 cells were transfected with siRNAs against targeted host genes or scrambled siRNA (NC). At 48 h p.t., cells were superinfected with SFTSV at an MOI of 1 and then collected at 48 h p.i. Viral protein levels were analyzed by Western blotting. (B) HEK 293 cells were cotransfected with plasmids expressing NSs or G proteins and siRNAs against targeted host genes or scrambled siRNA (NC). At 48 h p.t., the cells were collected and viral/host proteins were subjected to Western blot analyses.

We further investigated the roles of the three major UPR signaling pathways in determining the expression levels of individual viral proteins. In the case of SFTSV infection, intracellular expression levels of NP, NSs, RdRP, and GP were lower in PERK or ATF6 knockdown cells but not in XBP1 knockdown cells (Fig. 6A), which was in accordance with the above results that knockdown of ATF6 or PERK led to reduced virus replication and production (Fig. 4C and D). Moreover, the intracellular protein levels of GP decreased much more significantly than those of other viral proteins when the ATF6 or PERK signaling pathways were impaired (Fig. 6A), suggesting that GP was specifically downregulated. To further investigate the impact of the ATF6 and PERK pathways on GP expression, HEK 293 cells were first transfected with target siRNAs or scrambled siRNA and then transfected with plasmids expressing GP or NSs as a control at 24 h post-siRNA transfection. At 48 h post-plasmid transfection, HEK 293 cells were collected, and the intracellular levels of the two viral proteins were detected by Western blot analysis. As shown in Fig. 6B, knockdown of ATF6 or PERK resulted in decreased protein levels of intracellular GP but not of NSs. In contrast, neither GP nor NSs expression was influenced by the knockdown of XBP1. This result further confirmed that the proper expression of GP either with or without SFTSV infection is dependent on the ATF6 and PERK pathways.

### UPR signaling is also activated by infection with other closely related phleboviruses.

**FIG 7:**
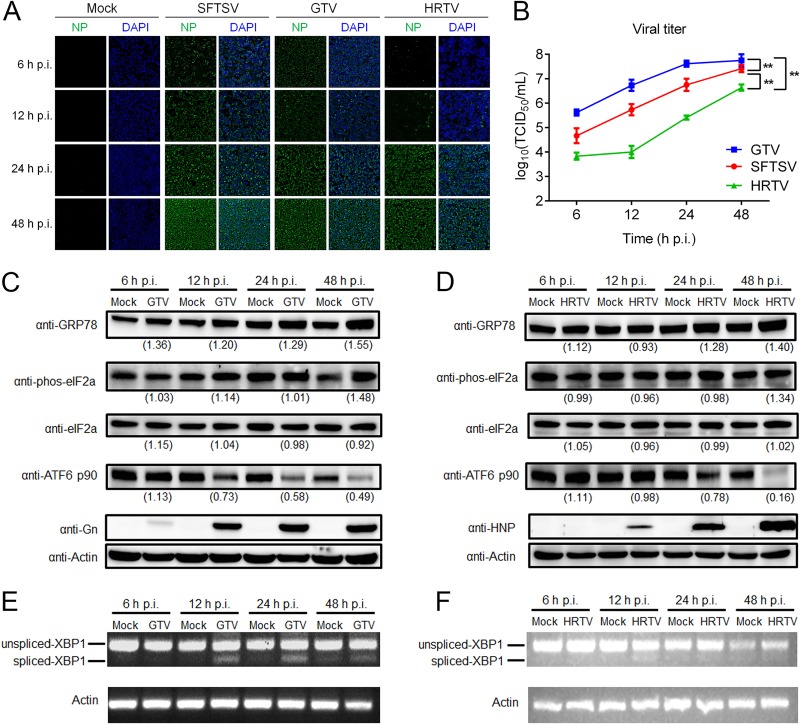
GTV and HRTV infection activate three branches of the UPR. (A) HEK 293 cells were infected by GTV, HRTV, or SFTSV, and then, at the indicated time intervals, the cells were fixed, and the intracellular level of NP was monitored by using immunofluorescence. (B) The supernatants of infected cells were also collected, and viral titers were measured by determining the TCID_50_. All experiments were performed at least three times, and values represent means ± the SDs from three replicates. ***, *P* < 0.001 (Fisher LSD tests). GTV (C)- or HRTV (E)-infected HEK 293 cells were collected at the indicated time intervals, and total proteins were extracted and subjected to Western blot analyses for GRP78, ATF6 p90, phos-eIF2α (Ser51), total eIF2α, Gn, NP, and the internal control actin. Anti-SFTSV Gn (anti-Gn) and anti-HRTV NP (anti-HNP) were used to detect GTV GP and HRTV NP, respectively. (D and F) RNA samples from the cells described above were also analyzed for spliced *XBP1* mRNA by using reverse transcription-PCR. The intensity of protein band was measured by ImageJ_v1.8.0. For each time point, the protein intensity was first normalized to actin and then normalized to the corresponding mock-treated group.

We next explored whether activation of the UPR is a unique feature for SFTSV infection or a common mechanism shared by some genetically closely related phleboviruses. As mentioned earlier, HRTV and GTV are two newly identified tickborne phleboviruses and are phylogenetically closely related to SFTSV (5, 7). We investigated the UPR responses in HEK 293 cells, which are also permissive to both HRTV and GTV infection (Fig. 7A) (7). The infection rates represented by the expression of NP (Fig. 7A) and the virus titers at different time points (Fig. 7B) showed that among the three viruses, the infectivity of GTV is the highest, while the infectivity of HRTV is the lowest in HEK 293 cells (*P* < 0.001). In GTV-infected HEK 293 cells, GRP78 was upregulated, suggesting that the UPR was activated (Fig. 7C). We found that decrease of ATF6 p90 protein (Fig. 7C) and splicing of *XBP1* mRNA began at 12 h p.i. (Fig. 7E), while the phosphorylation level of eIF2α protein increased obviously at 48 h p.i. (Fig. 7C), indicating that GTV can activate all three branches of the UPR. Similarly, in HRTV-infected HEK 293 cells, an increase in phosphorylation level of eIF2a protein began to be detected at 48 h p.i., while a decrease in ATF6 p90 protein began to be detected at 24 h p.i. (Fig. 7D), and a weak increase in the spliced *XBP1* mRNA was detected at 12 h p.i. (Fig. 7F), suggesting that infection of HRTV could also activate the three branches of the UPR. These data suggest that activation of the UPR may represent a common feature for phleboviruses, at least for SFTSV-related virus groups.

## DISCUSSION

Newly emerging phlebloviruses, such as SFTSV, HRTV, HRGV, etc., pose a serious threat to public health. Currently, there are no FDA-approved drugs or vaccines to combat phlebovirus infection, and this is in part due to a lack of comprehensive understanding of the molecular interactions occurring between phleboviruses and host cells. Although loss-of-function-based screenings at a whole-genome scale have been performed on different phleboviruses, including Rift Valley fever virus (RVFV) (9, 27), Uukuniemi virus (UUKV) (28), and SFTSV (9, 24), a global map showing how phleboviruses regulate and manipulate host biological processes for viral infection is still unavailable.

In the present study, to identify host proteins involved in the SFTSV replication process and decipher how virus infection affects biological processes of host cells, a quantitative proteomic analysis of SFTSV-infected cells was performed at 6, 12, 24, and 48 h p.i. A total of 5,606 host proteins were quantified, with 433 being differentially regulated, accounting for 7.7% of the quantified host proteins (Table S2). Among these, 310 upregulated proteins were identified, with ∼90% being enriched at the late stage of infection (48 h p.i.). Among these, only FN1 was upregulated across all time points, whereas 7 proteins were upregulated across three time points, 12 proteins were upregulated at two time points (Table S2). The other 123 proteins were found to be downregulated, and they were not enriched at any infection time point (Fig. 1D). Although a relatively high MOI was used in this study, we did not observe an obvious CPE or apoptosis in HEK 293 cells over a 48-h infection course (Fig. 1B). A previous report also demonstrated that no cell death or apoptosis was induced by SFTSV in monocyte THP-1 cells (29). Our proteomic data reflected that SFTSV infection cause a modest influence on host protein levels, which may partially explain the mild CPE caused by this virus.

Previous large-scale analyses of phlebovirus and host interactions via a “loss of function” strategy identified hundreds of host proteins that could affect phlebovirus infection (9, 27, 28, 30). Among these proteins, 44 were identified as being differentially regulated by SFTSV infection in this study (Table S3), suggesting that these proteins may play roles in the phlebovirus life cycle by changing protein levels via local synthesis and degradation. Among these proteins, two have been functionally characterized in other phleboviruses. ER chaperone GRP78 was upregulated in our study, and a previous siRNA screening study indicated that knockdown of GRP78 can inhibit the replication of UUKV (28); meanwhile, knockdown of NF-κB essential modulator (NEMO), another upregulated protein identified in our study, facilitated the replication of UUKV (28).

**FIG 8:**
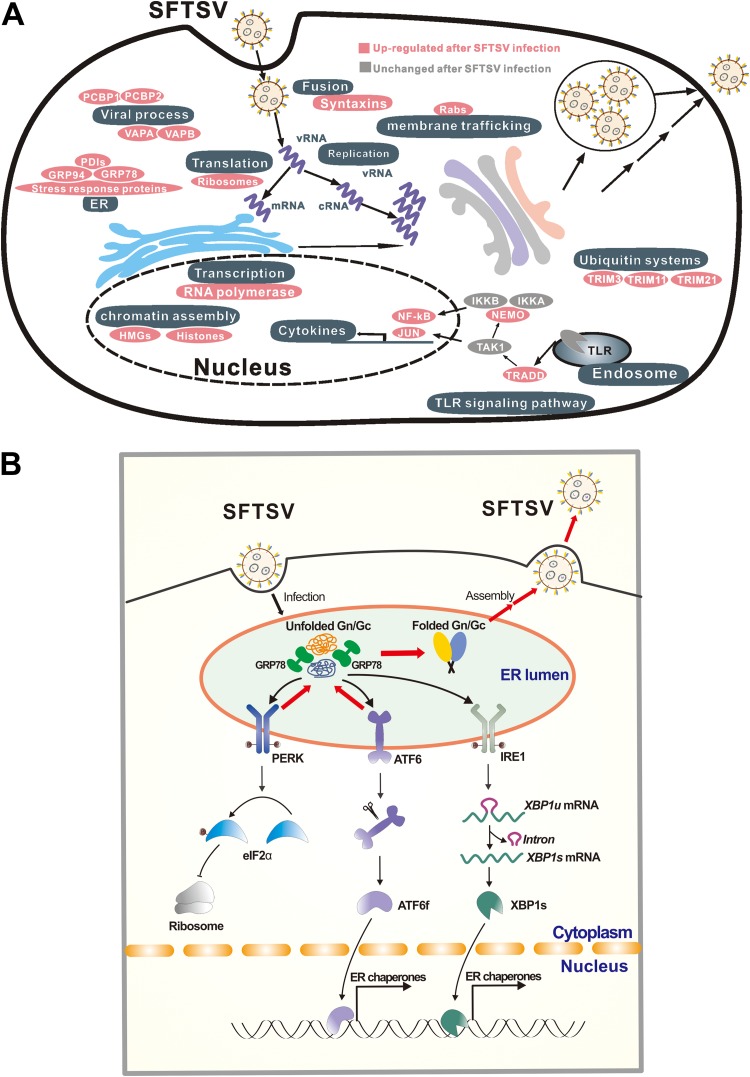
Proposed model for the UPR and other cellular responses regulated by SFTSV infection. (A) Global host cellular protein responses to SFTSV infection. According to the results of the quantitative proteomic analysis, proteins or protein complexes specifically regulated by SFTSV infection are sorted and aligned according to their biological functions. (B) Proposed interaction model between host UPR and SFTSV infection. SFTSV infection produces large amounts of unfolded GP in the ER, which activates all three main branches of the UPR. Among these, the ATF6 and PERK pathways facilitate proper folding of GP and thus favor SFTSV replication.

To better understand how SFTSV infection affects biological processes of host cells, a cellular response map was created, in which regulated proteins are sorted and aligned according to their biological functions. As shown in Fig. 8A, many biological processes and protein complexes are apparently regulated as a result of SFTSV infection. First, these processes and protein complexes include host proteins that may be involved in SFTSV replication cycle. For example, syntaxins (STX3 and 7), the small Rab GTPases (RabL3, Rab1A, Rab32, and Rab35), VAPA/VAPB are well-known cellular factors that participate vesicle trafficking, membrane fusion, protein complex assembly. Poly(C) binding proteins (PCBP) 1 and 2 facilitate viral replication of EV71, porcine reproductive and respiratory syndrome virus (31, 32). Second, these processes and proteins include the Toll-like receptor (TLR) signaling pathway. Although TLRs are generally expressed at low levels in HEK 293 cells, multiple modulators of the TLR signaling pathway were upregulated in SFTSV-infected cells, including NEMO, TRADD, and JUN (Fig. 8A and Table S2), suggesting that the TLR signaling pathway may be activated in SFTSV-infected HEK 293 cells. In SFTSV-infected patients, elevated proinflammation cytokines, including interleukin-1β (IL-1β), IL-18, and RANTES, were observed, suggesting SFTSV infection activates the production of proinflammation cytokines (33). A recent study of SFTSV-infected mice indicated that the TLR signaling pathway is essential for the production of type I interferon (IFN-I) and inflammatory cytokines *in vivo*, and enhanced production of multiple inflammatory cytokines and chemokines may trigger the lethal SFTS (34). Third, these processes and proteins include the ubiquitin system. Multiple host proteins involved in the ubiquitin system were differentially regulated after SFTSV infection (Fig. 8A), including E3 ubiquitin ligase TRIM11 (tripartite motif-containing protein 11) and TRIM21, both of which were upregulated at 48 h p.i. (Table S2). Another protein involved in the ubiquitin system, OTULIN (OTU domain-containing deubiquitinase with linear linkage specificity), was upregulated. OTULIN is an essential negative regulator of inflammation (35, 36). Fourth and finally, these processes and proteins include ER stress. Gene ontology (GO) analysis indicated that the proteins involved in ER stress and the UPR were also upregulated at both 24 and 48 h p.i. Particularly, GRP78, the most notable marker and important chaperon of UPR, was upregulated at 12, 24, and 48 h p.i., suggesting that the UPR was apparently induced by SFTSV infection (Fig. 2C and D).

Next, we performed an in-depth investigation of the interactions between SFTSV and the three classical branches of host UPR, including PERK, ATF6, and IRE1. The result showed that SFTSV infection could activate all the three branches of UPR (Fig. 3); however, only PERK and ATF6 pathways were found to play important roles in SFTSV infection (Fig. 4). PERK can sense an excess of unfolded proteins in the ER and subsequently phosphorylates the eIF2α, attenuating global protein synthesis (26). Multiple viruses have been reported that can affect eIF2α phosphorylation, including human cytomegalovirus (37), dengue virus (26), West Nile virus (WNV) (38), ZIKV (39), Junín virus, and Machupo virus (40). WNV (16, 38), CSFV (15), and TGEV (18) can activate PERK-mediated eIF2α phosphorylation (26). However, the activation of eIF2α phosphorylation upon TGEV infection inhibited TGEV replication by suppressing protein translation and promoting IFN-I production (18). Although eIF2α phosphorylation was induced by SFTSV (Fig. 3C), we did not detect a significant decrease in the overall protein levels. Therefore, the mechanism for how SFTSV promotes productive infection via phosphorylation of eIF2α and the capacity of SFTSV to translate in an eIF2α-independent manner are currently unclear. In addition, although the PERK-eIF2α pathway was activated by SFTSV infection (Fig. 3C), we could not exclude the possibility that phosphorylation of eIF2α may occur in the context of the UPR by other kinases, such as heme-regulated inhibitor that responds to heme deprivation, general control non-derepressible-2 (GCN2) that responds to amino acid deprivation, and protein kinase R (PKR), which is activated by double-stranded RNA (10, 41). Activation of the ATF6 pathway targets ATF6 from the ER to the Golgi compartment, where it is proteolytically cleaved (42), and ATF6 further translocates into the nucleus to activate the expression of ER chaperones (10) (Fig. 8B). WNV_KUN_ infection can activate the ATF6 pathway to facilitate replication and immune evasion (16). Acute lymphocytic choriomeningitis (LCMV) infection selectively induces the ATF6 pathway, which is likely beneficial for virus replication and cell viability (17). Flaviviruses have evolved to activate the IRE1-XBP1 arm of the UPR, and this may expand both the time and the space available for flavivirus replication (43). Here, we found that knockdown of key UPR sensors, PERK or ATF6, reduced viral titers (Fig. 4). Potentially, the UPR may extend the lifespan of an infected cell, thereby increasing the levels of progeny virus.

We also investigated whether activation of the UPR is a common mechanism shared by the SFTSV-related phleboviruses, HRTV and GTV. Distinct effects on UPR induction have been observed in genetically related viruses. For example, in the cases of alphaviruses, Semliki Forest virus (SFV) infection can activate the UPR (44), while Chikungunya virus infection suppresses the UPR (45). Differing phenomena have been found even for the same virus. For example, infection with WNV NY-99 strain activates all three pathways of the UPR (38), while infection with the WNV_KUN_ strain activates the ATF6 and XBP-1 pathways but not the PERK pathway (16), and this may be due to differences in the viral strains and/or cell lines used. Our results suggest that activation of ER stress and UPR is not confined to SFTSV infection but seems to be conserved in some genetically related viruses, although the detailed roles of host UPR in the HRTV and GTV life cycles require further investigation. However, we also noticed slight differences in the extent and/or time course of UPR activation by SFTSV, HRTV, or GTV, and this may be due to the different cell sensitivity to the three distinct phleboviruses (Fig. 7A and B), resulting in different viral replication and protein expression levels.

Virus surface glycoproteins or membrane proteins have been reported as one of the inducers of the UPR in some viruses, such as the G protein of LCMV (17), spike protein of severe acute respiratory syndrome-associated coronavirus (SARS-CoV) (46), and hydrophobic proteins of flaviviruses (16, 43, 47). Here, we identified GP, but not the other viral proteins (RdRP, NP, or NSs) of SFTSV as the inducer of the UPR (Fig. 5). Activation of the UPR by SFTSV GP may be caused by accumulation of unfolded or misfolded proteins in the ER, since GP alone localizes in the ER, as well as in the ER-Golgi complex, and is responsible for recruiting RdRP and NP into these compartments during virus infection (48). Aggregated GP may modify membrane permeability of the ER, in turn altering ion homeostasis (10, 49). In addition, viral glycoproteins generally contain abundant posttranslational modifications (PTMs), such as glycosylation and disulfide-bond formation, and are closely associated with ER stress and the UPR, probably via competing with host protein for modifications (50). Crystal structure information indicated that SFTSV GP contains five N-glycosylation sites and is stabilized by 21 disulfide bonds (51, 52). The PTMs in SFTSV GP may be one of the inducers triggering ER stress and the UPR, which requires further study. As indicated in Fig. 8B, SFTSV infection produces large amounts of GP in the ER; this induces ER stress and activates all three main branches of the UPR. The ATF6 and PERK pathways are important for maintaining intracellular protein levels (Fig. 6) and probably correct folding of GP; thus, these may benefit SFTSV proliferation.

Taken together, this study, for the first time to our knowledge, provides a global map for host cellular responses to SFTSV infection. By examining differentially regulated host proteins, we demonstrated that SFTSV infection could induce the UPR, which further favored SFTSV replication. Furthermore, a critical role of SFTSV GP in activation of the UPR was elucidated, and activation of ER stress and the UPR was implicated as a common feature shared by SFTSV-related phleboviruses, at least HRTV and GTV. In addition, many other important host biological processes highlighted by this research provide potential host targets for basic research, as well as anti-SFTSV drug development.

## MATERIALS AND METHODS

### Cells and viruses.

HEK 293 cells and Vero cells were obtained from the China Center for General Virus Culture Collection (CCGVCC) and grown in Dulbecco modified Eagle medium (DMEM; Gibco) supplemented with 10% fetal bovine serum (FBS; Gibco) at 37°C with 5% CO_2_. SFTSV WCH-2011/HN/China/isolate 97 (53), Heartland virus isolate Patient1 (5), and GTV strain DXM (7) were obtained from the CCGVCC and propagated in Vero cells in a biosafety level 3 laboratory.

### Viral infectivity analysis.

To analyze the one-step growth curve of SFTSV in HEK 293 cells, SFTSV was inoculated in HEK 293 cells at an MOI of 5 TCID_50_/cell. After 1 h of attachment, the supernatant was replaced with fresh cell culture medium. The tissue culture supernatant of the infected HEK 293 cells was collected at 3, 6, 12, 24, 36, 48, and 72 h p.i., and virus titers were determined by an EPDA (monitoring the expression of NP by immunofluorescence microscopy) as previously described (54).

To compare the infectivity of SFTSV, GTV and HRTV, the three viruses were used to infect HEK 293 cells at an MOI of 5 TCID_50_/cell, and virus titers were determined at 6, 12, 24, and 48 h p.i. by EPDAs. The infection experiment was performed in triplicate, and virus titers were analyzed using two-way analysis of variance (SPSS, Inc.) with virus type and time as factors. If significant effects were found, difference of titers between two viruses was evaluated by Fisher least significant difference (LSD) tests.

### iTRAQ labeling and LC-MS/MS analysis.

HEK 293 cells were infected with SFTSV at an MOI of 5 or mock infected. At 6, 12, 24, and 48 h p.i., cells were harvested, and the extracted proteins were reduced with 10 mM dithiothreitol and alkylated with 40 mM iodoacetomide before being digested with trypsin (Promega). The digested peptides were desalted with a SepPak C_18_ cartridge (Waters) and dried using a SpeedVac (Thermo). Three independent biological replicates were performed.

For iTRAQ labeling, 100 μg of peptides from SFTSV- or mock-infected cells was resuspended in iTRAQ dissolution buffer, and then different iTRAQ reagents (SCIEX) were added. After being incubated in room temperature for 1 h, equal amounts of labeled peptides were mixed and desalted with a SepPak C_18_ cartridge. The mixed peptides were fractionated using strong-cation exchange as previously described (55). The fractionated peptides were dried by SpeedVac and stored at –80°C.

LC-MS/MS analysis was performed on a quadrupole-time of flight LC-MS/MS mass spectrometer (TripleTOF 5600+; SCIEX) equipped with a nanospray source. Peptides were first loaded onto a C_18_ trap column (Agilent Technologies) and then eluted into a C_18_ analytical column (Eksigent). For MS/MS analysis, each scan cycle consisted of one full-scan mass spectrum, followed by 20 MS/MS events. Mass spectra were extracted by Peakview v2.0 (SCIEX).

### MS data analysis.

Three independent biological replicates were performed, and peptides from three biological replications were analyzed by LC-MS/MS independently. MS spectra were submitted to ProteinPilot v5.0.1 (SCIEX) to perform peptide identification and quantification. The UniProt_Human database was used. Search parameters were as follows: sample type, iTRAQ 8plex (peptide labeled); cysteine alkylation, iodoacetamide; digestion, trypsin; miss cleavages tolerance, 2; fixed modification, carbamidomethyl Cys; variable modification, none; MS1 initial mass error tolerance value, 0.05 Da; MS2 initial mass error tolerance value, 0.1 Da; and instrument, TripleTOF 5600. The false discovery rate (FDR) analysis in ProteinPilot uses a “decoy database searching” strategy, the FDRs of ProteinPilot search results were all set as lower than 1% at the protein level, and only peptides with confidence scores of >95% were used. In each replicate, protein ratio was calculated from the weighted average ratios of each peptide, with peptide intensity as the weight. The protein ratio values used for bioinformatics analysis were the weighted averages of the three biological replicates, while the *P* value for protein ratio was calculated and further corrected with multiple Bonferroni correction (Table S1). The cutoff for differentially regulated proteins was set as described in a previous study (56, 57). Briefly, the Gaussian distribution of protein ratios was analyzed, and values deviating from the mean of the normally distributed data by 3.3 standard deviations (SD) were considered cutoff values. Only proteins meeting the following two criteria were considered differentially regulated: (i) ratios > upregulated or < downregulated cutoff values and (ii) corrected *P* value for a protein ratio of < 0.05.

### GO analysis.

To perform GO analysis, differentially regulated proteins were submitted to DAVID (https://david.ncifcrf.gov/) (58), with all quantified proteins in this study being set as the background. Proteins were classified into different categories based on their roles in biological processes, and a statistical overrepresentation test was performed. *P* values were assessed with a binomial test and corrected for multiple testing using a Bonferroni procedure. Only categories with a *P* value of <0.05 were considered over- or underrepresented.

### Western blot analysis.

Mouse monoclonal antibodies against β-actin (ProteinTech, China) and GRP78 (HuaBio, China), and rabbit polyclonal antibodies against PERK, ATF6, XBP1, and HSP90AB1 (ProteinTech, China) and eIF2α and phospho-eIF2α (Ser51; Cell Signaling Technology) were purchased from the indicated manufacturers.

Rabbit or mouse sera against SFTSV NSs (anti-NSs), NP (anti-NP), GP (anti-Gn), RdRP (anti-RdRP), and HRTV NP (anti-HNP) were used to probe the corresponding proteins expressed in HEK 293 cells (59). The antibody against STFSV GP (anti-Gn) was used to detect GTV GP, since GTV GP share 79.4% amino acid identity with SFTSV GP (7). For Western blot analysis, protein samples were subjected to SDS-PAGE and then transferred onto polyvinylidene difluoride membranes (Millipore). After being blocked with 5% bovine serum albumin in Tris-buffered saline/Tween 20, the membrane was probed with primary antibodies and then with horseradish peroxidase-conjugated secondary antibodies. Protein bands were detected by an enhanced chemiluminescence kit (Thermo Fisher Scientific).

### PBMC preparation.

Mouse PBMCs were isolated from blood samples by density gradient centrifugation method using Histopaque (Sigma). Briefly, the blood was layered on lymphocyte separation medium gently in the ratio of 1:1 and subjected to centrifugation at 100 × *g* for 30 min. The white layer representing PBMCs was aspirated out gently and transferred aseptically into sterile centrifuge tubes. The suspension of cells was then washed and cultured in DMEM supplemented with 20 mM l-glutamine, 10% FBS, 100 U/ml of penicillin, and 100 μg/ml of streptomycin.

### Quantitative real-time PCR.

Cells were harvested, and total cellular mRNA was extracted using TRIzol reagent (Promega). mRNA was then subjected to reverse transcription using a Moloney murine leukemia virus (MMLV) reverse transcriptase (Promega). Quantitative RT-PCR was performed using specific primers for targeting genes with SYBR Premix Ex Taq (Applied Biosystems) on an Applied Biosystems 7500 real-time PCR system.

### *XBP1* splicing assay.

SFTSV-, HRTV-, GTV-, or mock-infected cells were harvested at different time points, and total cellular RNA was extracted with TRIzol reagent (Promega). cDNA was synthesized using a Moloney murine leukemia virus reverse transcriptase (Promega). *XBP1* cDNA was amplified using primers (5′-CATGGCCTTGTAGTTG-3′ and 5′-CTGGGTCCACCAAGTTGT-3′) containing the IREI splicing site (17). PCR products of ∼270 and ∼244 bp, representing unspliced and spliced *XBP1*, respectively, were separated on 2% agarose gels.

### siRNA transfection.

A total of 2 × 10^5^ HEK 293 cells preseeded in 24-well plates were transfected with 20 pmol of siRNA using Lipofectamine RNAiMAX (Invitrogen) according to the manufacturer’s instructions. In a parallel experiment, scrambled siRNA was included as a control. At 24 h p.t., cells were collected and subjected to further analyses. All siRNA oligonucleotides used in the study were synthesized by GenePharma (Suzhou, China), and the sequences were as follows (5′–3′): siPERK#1, GUUGUGCUAGCAACCCUAAUA; siPERK#2, GGAACGACCUGAAGCUAUAAA; siATF6#1, GCAGCAACCAAUUAUCAGUUU; siATF6#2, CCACCCAUAACAAGACCACAA; siXBP1#1, GCCUGUCUGUACUUCAUUCAA; and siXBP1#2, AGAUCGAAAGAAGGCUCGAAU.

### Plasmid transfection.

HEK 293 cells (2 × 10^5^) preseeded in 24-well plates were transfected with plasmids encoding SFTSV *NP*, *NSs*, *GP*, and *RdRP* genes, respectively, using Lipofectamine 2000 (Invitrogen) according to the manufacturer’s instructions. In parallel experiments, empty vector and (or) plasmid expressing enhanced green fluorescent protein were included as controls. At 24 h p.t., the cells were collected and subjected to further analyses.

### MTT assay.

HEK 293 cells were infected with SFTSV (MOI = 5) and harvested at 24, 48, and 72 h p.i., or HEK 293 cells were treated with the desired concentrations of siRNAs for 24 h. Then, MTT (Sigma-Aldrich) was added at a final concentration of 5 mg/ml. The cells were incubated at 37°C for 4 h, and the supernatant was removed. Then, 50 μl of dimethyl sulfoxide was added to each well, and the emitted light at 492 nm was measured with a Thermo Multiskan enzyme-linked immunosorbent assay reader (Thermo, Waltham, MA).

## Supplementary Material

Supplemental file 1

Supplemental file 2

Supplemental file 3
