# Supplementary material for: Quantitative Proteomic Analysis Reveals Unfolded-Protein Response Involved in Severe Fever with Thrombocytopenia Syndrome Virus Infection
Source: J Virol. 2019 May 1;93(10):e00308-19. doi: 10.1128/JVI.00308-19 (PMC6498065; doi:10.1128/JVI.00308-19)
Supplement: Supplemental file 3 [file JVI.00308-19-s0003.pdf]

No.	Gene name	Proteins	6 h p.i.		12 h p.i.		24 h p.i.		48 h p.i.		Virus	Strategy	Effect	Reference
			Log$_2$(ratio)	*P*- value	Log$_2$(ratio)	*P*- value	Log$_2$(ratio)	*P*- value	Log$_2$(ratio)	*P*- value				
1	ACAD8	Isobutyryl-CoA dehydrogenase, mitochondrial	0.32	0.58	-0.52	0.01	0.02	0.95	-0.15	0.29	**RVFV**	siRNA	pro-viral	(1)
2	ADAT2	tRNA-specific adenosine deaminase 2	-0.45	-	0.29	-	0.03	-	0.49	-	**RVFV**	siRNA	pro-viral	(1)
3	AHNAK	Neuroblast differentiation-associated protein AHNAK	-0.02	0.13	-0.11	0.05	-0.02	0.24	0.37	0.01	**RVFV/UUKV**	siRNA	pro-viral	(1)/(2)
4	AMFR	E3 ubiquitin-protein ligase AMFR	0.44	0.11	0.15	0.57	0.36	0.48	0.18	0.21	**rVSV-ANDV G**	HAP	pro-viral	(3)
5	B3GALT6	Beta-1,3-galactosyltransferase 6	0.52	0.03	0.07	0.23	0.31	0.32	0.88	0.13	**RVFV**	HAP	pro-viral	(4)
6	BOP1	Ribosome biogenesis protein BOP1	-0.05	0.44	-0.01	0.81	0.09	0.15	0.43	0.02	**UUKV/rVSV-SFTSV G**	siRNA	pro-viral	(2)/(5)
7	CDS2	Phosphatide cytidylyltransferase 2	-0.26	0.31	-0.34	0.16	-0.48	0.01	0.34	0.35	**UUKV**	siRNA	pro-viral	(2)
8	COX16	Cytochrome c oxidase assembly protein COX16 homolog, mitochondrial	-0.23	-	-0.46	-	-0.53	-	0.43	-	**UUKV**	siRNA	pro-viral	(2)
9	CWC15	Spliceosome-associated protein CWC15 homolog	-0.08	0.27	-0.11	0.25	-0.02	0.70	0.41	0.05	**RVFV**	siRNA	anti-viral	(6)
10	DPH1	Diphthamide biosynthesis protein 1	0.12	0.59	0.10	0.60	0.10	0.46	0.38	0.02	**RVFV**	siRNA	pro-viral	(1)
11	EBNA1BP2	Probable rRNA-processing protein EBP2	0.09	0.05	-0.01	0.88	0.07	0.26	0.38	0.01	**RVFV**	siRNA	anti-viral	(6)
12	FKBP2	Peptidyl-prolyl cis-trans isomerase FKBP2	-0.09	0.17	-0.06	0.53	0.07	0.09	0.41	0.01	**RVFV**	siRNA	pro-viral	(1)
13	GGA1	ADP-ribosylation factor-binding protein GGA1	-0.07	0.40	-0.02	0.74	-0.05	0.79	0.37	0.00	**UUKV**	siRNA	pro-viral	(2)
14	GRP78	78 kDa glucose-regulated protein	-0.05	0.14	0.37	0.01	0.51	0.00	0.70	0.00	**UUKV**	siRNA	pro-viral	(2)
15	NEMO	NF-kappa-B essential modulator	-0.11	0.41	-0.07	0.40	-0.12	0.15	0.37	0.00	**UUKV**	siRNA	anti-viral	(2)
16	MAU2	MAU2 chromatid cohesion factor homolog	-0.08	0.49	0.04	0.66	-0.46	0.02	0.08	0.52	**UUKV**	siRNA	pro-viral	(2)
17	MBTPS2	Membrane-bound transcription factor site-2 protease	0.03	0.92	0.26	0.54	-0.62	0.08	0.10	0.63	**UUKV**	siRNA	pro-viral	(2)
18	MED7	Mediator of RNA polymerase II transcription subunit 7	-0.06	0.75	0.00	0.99	0.70	0.23	0.54	0.06	**UUKV**	siRNA	pro-viral	(2)
19	NMT1	Glycylpeptide N-tetradecanoyltransferase 1	0.02	0.32	-0.08	0.13	0.12	0.03	0.37	0.02	**RVFV**	siRNA	anti-viral	(6)
20	POLR2J	DNA-directed RNA polymerase II subunit RPB11-a	-0.08	0.60	0.04	0.16	0.07	0.53	0.38	0.04	**RVFV**	siRNA	anti-viral	(6)
21	QPRT	Nicotinate-nucleotide pyrophosphorylase [carboxylating]	0.09	-	0.28	-	0.03	-	0.39	-	**UUKV**	siRNA	pro-viral	(2)
22	RABGGTB	Geranylgeranyl transferase type-2 subunit beta	0.09	0.65	0.00	0.98	-0.16	0.03	0.46	0.01	**RVFV**	siRNA	pro-viral	(1)
23	RPL11	60S ribosomal protein L11	0.07	0.22	-0.14	0.14	-0.04	0.18	0.48	0.01	**RVFV**	siRNA	ribosome	(6)

24	RPL19	60S ribosomal protein L19	-0.03	0.57	-0.05	0.27	-0.12	0.06	0.39	0.01	**RVFV**	siRNA	ribosome	(6)
25	RPL23A	60S ribosomal protein L23a	0.03	0.49	-0.14	0.04	0.01	0.90	0.51	0.01	**RVFV**	siRNA	ribosome	(6)
26	RPL31	60S ribosomal protein L31	0.14	0.11	-0.03	0.69	-0.10	0.12	0.37	0.00	**RVFV**	siRNA	ribosome	(6)
27	RPL34	60S ribosomal protein L34	0.06	0.16	-0.09	0.02	0.02	0.59	0.57	0.01	**RVFV**	siRNA	ribosome	(6)
28	RPL35	60S ribosomal protein L35	0.04	0.72	-0.03	0.80	-0.14	0.09	0.44	0.02	**UUKV**	siRNA	anti-viral	(2)
29	RPL35A	60S ribosomal protein L35a	0.00	0.98	-0.17	0.07	-0.04	0.44	0.37	0.03	**RVFV**	siRNA	ribosome	(6)
30	RPL36	60S ribosomal protein L36	-0.08	0.37	-0.12	0.01	-0.05	0.55	0.38	0.01	**RVFV**	siRNA	ribosome	(6)
31	RRM2	Ribonucleoside-diphosphate reductase subunit M2	0.08	0.37	0.09	0.04	0.05	0.69	0.44	0.01	**RVFV**	siRNA	anti-viral	(6)
32	SAFB2	Scaffold attachment factor B2	-0.07	0.38	-0.01	0.81	-0.09	0.29	0.38	0.03	**UUKV**	siRNA	pro-viral	(2)
33	SLC39A7	Zinc transporter SLC39A7	0.10	-	-0.39	-	0.67	-	0.54	-	**UUKV**	siRNA	pro-viral	(2)
34	SLC44A1	Choline transporter-like protein 1	0.11	-	-0.09	-	0.45	-	-0.06	-	**RVFV**	siRNA	anti-viral	(6)
35	SNCG	Gamma-synuclein	-0.33	-	0.26	-	-0.81	-	0.35	-	**RVFV**	siRNA	pro-viral	(1)
36	TMEM87A	Transmembrane protein 87A	-0.09	-	-0.03	-	-0.46	-	0.37	-	**rVSV-SFTSV**	HAP	pro-viral	(5)
37	TNRC6B	Trinucleotide repeat-containing gene 6B protein	-0.07	-	-0.21	-	-0.08	-	0.46	-	**UUKV/rVSV-SFTSV**	siRNA	anti-viral/pro-viral	(2)/(5)
38	TPT1	Translationally-controlled tumor protein	-0.12	0.01	-0.12	0.28	0.03	0.55	0.40	0.04	**UUKV**	siRNA	pro-viral	(2)
39	TRMT61A	tRNA (adenine(58)-N(1))-methyltransferase catalytic subunit TRMT61A	0.09	0.15	-0.04	0.74	0.15	0.18	0.41	0.01	**UUKV**	siRNA	anti-viral	(2)
40	TSC22D4	TSC22 domain family protein 4	-0.05	0.50	0.03	0.78	0.22	0.21	0.72	0.02	**RVFV**	siRNA	pro-viral	(1)
41	VPS13D	Vacuolar protein sorting-associated protein 13D	0.18	-	0.40	-	-0.48	-	0.39	-	**rVSV-SFTSV G**	HAP	pro-viral	(5)
42	YBX1	Nuclease-sensitive element-binding protein 1	-0.01	0.79	-0.14	0.20	-0.12	0.08	0.46	0.04	**UUKV**	siRNA	pro-viral	(2)
43	ZIC5	Zinc finger protein ZIC 5	0.26	0.34	-0.19	0.60	-0.68	0.17	0.28	0.53	**rVSV-ANDV G**	HAP	pro-viral	(3)
44	ZMYND8	Protein kinase C-binding protein 1	0.09	0.24	0.14	0.32	0.09	0.30	0.60	0.02	**UUKV**	siRNA	pro-viral	(2)

Table S3. 44 regulated proteins identified in this study have been reported in previous study that can affect bunyavirus infection.

The protein ratio values used are the weighted averages of the three biological replicates, and was present as $\log_2$[$\log_2$(protein ratio)].

P value for protein ratio was calculated and further corrected with multiple Bonferroni correction.

Virus: viruses used in those studies.

siRNA: siRNA screenging; HAP: Haploid screen.

Pro-viral: protein that can promote replication of virus; anti-viral: protein that can inhibit replication of virus; ribosome: ribosome protein.

Reference

1. Harmon B, Bird SW, Schudel BR, Hatch AV, Rasley A, Negrete OA. 2016. A Genome-Wide RNA Interference Screen Identifies a Role for Wnt/beta-Catenin Signaling during Rift Valley Fever Virus Infection. J Virol 90:7084-7097.
2. Meier R, Franceschini A, Horvath P, Tetard M, Mancini R, von Mering C, Helenius A, Lozach PY. 2014. Genome-wide small interfering RNA screens reveal VAMP3 as a novel host factor required for Uukuniemi virus late penetration. J Virol 88:8565-8578.
3. Petersen J, Drake MJ, Bruce EA, Riblett AM, Didigu CA, Wilen CB, Malani N, Male F, Lee FH, Bushman FD, Cherry S, Doms RW, Bates P, Briley K, Jr. 2014. The major cellular sterol regulatory pathway is required for Andes virus infection. PLoS pathogens 10:e1003911.
4. Riblett AM, Blomen VA, Jae LT, Altamura LA, Doms RW, Brummelkamp TR, Wojcechowskyj JA. 2015. A Haploid Genetic Screen Identifies Heparan Sulfate Proteoglycans Supporting Rift Valley Fever Virus Infection. J Virol 90:1414-1423.
5. Drake MJ, Brennan B, Briley K, Jr., Bart SM, Sherman E, Szemiel AM, Minutillo M, Bushman FD, Bates P. 2017. A role for glycolipid biosynthesis in severe fever with thrombocytopenia syndrome virus entry. PLoS pathogens 13:e1006316.
6. Hopkins KC, McLane LM, Maqbool T, Panda D, Gordesky-Gold B, Cherry S. 2013. A genome-wide RNAi screen reveals that mRNA decapping restricts bunyaviral replication by limiting the pools of Dcp2-accessible targets for cap-snatching. Genes & development 27:1511-1525.
